# Plasma Components and Platelet Activation Are Essential for the Antimicrobial Properties of Autologous Platelet-Rich Plasma: An *In Vitro* Study

**DOI:** 10.1371/journal.pone.0107813

**Published:** 2014-09-18

**Authors:** Lorenzo Drago, Monica Bortolin, Christian Vassena, Carlo L. Romanò, Silvio Taschieri, Massimo Del Fabbro

**Affiliations:** 1 Laboratory of Technical Sciences for Laboratory Medicine, Department of Biomedical Science for Health, University of Milan, Milan, Italy; 2 Laboratory of Clinical Chemistry and Microbiology, IRCCS Galeazzi Orthopedic Institute, Milan, Italy; 3 Dental Clinic, IRCCS Galeazzi Orthopedic Institute, Milan, Italy; 4 Department of Biomedical, Surgical and Dental Sciences, University of Milan, Milan, Italy; 5 Center of Reconstructive Surgery and Osteoarticular Infection, IRCCS Galeazzi Orthopedic Institute, Milan, Italy; University Hospital of the Albert-Ludwigs-University Freiburg, Germany

## Abstract

Autologous platelet concentrates are successfully adopted in a variety of medical fields to stimulate bone and soft tissue regeneration. The rationale for their use consists in the delivery of a wide range of platelet-derived bioactive molecules that promotes wound healing. In addition, antimicrobial properties of platelet concentrates have been pointed out. In this study, the effect of the platelet concentration, of the activation step and of the presence of plasmatic components on the antimicrobial activity of pure platelet-rich plasma was investigated against gram positive bacteria isolated from oral cavity. The antibacterial activity, evaluated as the minimum inhibitory concentration, was determined through the microdilution two-fold serial method. Results seem to suggest that the antimicrobial activity of platelet-rich plasma against *Enterococcus faecalis*, *Streptococcus agalactiae*, *Streptococcus oralis* and *Staphylococcus aureus* is sustained by a co-operation between plasma components and platelet-derived factors and that the activation of coagulation is a fundamental step. The findings of this study may have practical implications in the modality of application of platelet concentrates.

## Introduction

Autologous platelet concentrates are successfully adopted in a variety of medical fields to stimulate hard and soft tissue healing and to control postoperative wound bleeding. The rationale for their use consists in the delivery of a wide range of growth factors and other bioactive molecules, stored in platelet α-granules and released upon platelet activation, that promote migration, proliferation and differentiation of the cells involved in wound healing and regenerative processes [1], [2]. In addition, anti-inflammatory properties of platelet concentrates have been pointed out, associated with a marked reduction of postoperative pain and swelling [3]–[5].

Platelet concentrates are also believed to exhibit antimicrobial properties. Recent studies have evaluated clinical and *in vitro* antibacterial activity of platelet concentrates, showing inhibitory effect against various microorganisms [6]–[20]. To date, the components responsible for the antimicrobial activity of platelet concentrates are poorly understood. In fact, platelet concentrates are a complex mixture of platelets, leukocytes and plasma, and the respective impact of the plasma and cellular components has not been studied in detail yet.

*Enterococcus faecalis* is a microorganism associated with different forms of periradicular disease, including primary extra-radicular and post-treatment persistent infections [21]. Such microorganism possesses the ability to survive the effects of root canal treatment and persist as a pathogen in the root canals and dentinal tubules of teeth. Implementing methods to effectively eliminate *E*. *faecalis* from the dental apparatus is a challenge.

*Streptococcus* strains are consistently present in the human oral cavity and are the major early colonizers of dental biofilm. Strains of many species of oral streptococci are generally commensal in humans. However, strains of other species, including those of *S. oralis* and *S. agalactiae*, may be more commonly associated with significant infections [22]–[23].

The oral cavity and oropharynx deserve greater attention as a reservoir of *Staphylococcus aureus.* Studies suggested that the presence of *S. aureus* in saliva was a significant risk factor for aspiration pneumonia [24], [25]. Oral *S. aureus* may also serve as a reservoir for cross-infection to other patients, as well as health care staff [26].

The aim of this study was to evaluate the antimicrobial effect of different platelet concentrations, of the activation step and of the presence of plasmatic components in a leukocyte-poor platelet concentrate (pure platelet-rich plasma, P-PRP) against *E. faecalis*, *S. agalactiae*, *S. oralis* and *S. aureus* strains isolated from oral cavity.

## Materials and Methods

### Donors

Blood samples were obtained from 20 healthy donors. All subjects were in general good health condition (ASA 1-2). No patient took antibiotics during the month before surgery, nor was under anticoagulant or immunosuppressive therapy. Written informed consent for participation in the study was obtained from all patients. The present research was performed within the guidelines of the Helsinki Declaration for biomedical research involving human subjects. The study was approved by the Review Board of the Galeazzi Orthopedic Institute.

### Blood collection and production of platelet concentrates

Fresh human whole blood from donors was processed using a laboratory centrifuge (Megafuge 1.0 R, Heraeus, Hanau, Germany) to obtain P-PRP, following Anitua's protocol [8]. Briefly, peripheral blood (4.5 ml) from each donor was taken by venipuncture into 5 ml blood-collecting tubes (Vacutainer, Franklin Lakes, New Jersey, U.S.A.) with 3.8% (wt/vol) trisodium citrate as anticoagulant. A pool was created with blood collected from all patients. Blood was centrifuged at 580 g for 8 min at room temperature. After centrifugation, three components were obtained: red blood cells at the bottom of the tube; a thin layer of leukocytes referred to as “buffy coat” in the middle; and plasma, which contains most of the platelets, at the top. The plasma fraction located above the red cell fraction, but not including the buffy coat, was collected (P-PRP).

To evaluate the quality of the P-PRP produced, platelet and leukocyte counts were performed using a hematology analyzer (XE-2100, Sysmex Europe, Norderstedt, Germany). According to Anitua *et al*., platelet concentration in P-PRP is at least twice the concentration in whole blood, while leukocyte concentration is consistently lower (<10^3^ white blood cells/µl) [8].

**Figure 1:**
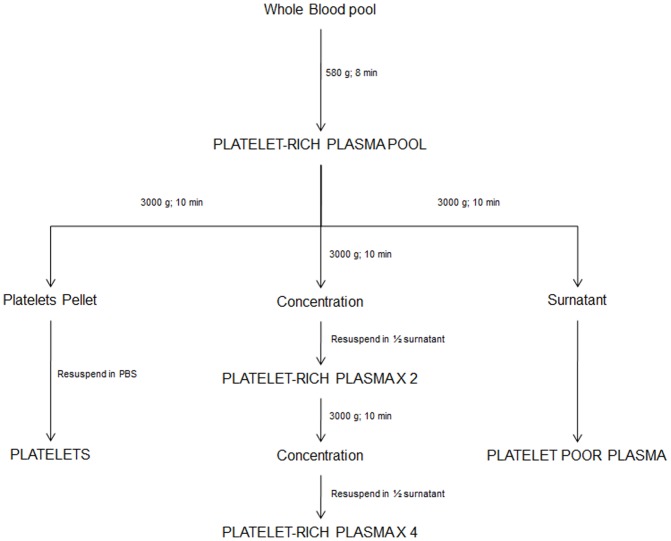
Experimental design of the study. The image illustrates the procedure used to obtain P-PRP, PPP, the pure platelet solution, 2-fold and 4-fold concentrated P-PRP. Starting from a whole blood pool, a P-PRP pool was obtained by centrifugation at 580 g for 8 minutes. Subsequently, P-PRP pool was divided into three equal aliquots and processed in order to obtain the different materials under evaluation in this study.

Subsequently, three equal aliquots of P-PRP were collected and processed as follows (Figure 1):

1. To prepare platelet-poor plasma (PPP), an aliquot of P-PRP was centrifuged at 3000 g for 10 minutes. The supernatant was recovered (PPP).
2. To obtain a pure platelet solution without plasmatic components, an aliquot of P-PRP was centrifuged at 3000 g for 10 minutes. The supernatant was removed, and the platelet pellet was resuspended in a volume of phosphate buffered saline (PBS; Sigma-Aldrich, St. Louis, Missouri, U.S.A.) equal to that of the supernatant removed.
3. To further increase platelet concentration, an aliquot of P-PRP was centrifuged at 3000 g for 10 minutes. The supernatant was recovered, and the platelet pellet was resuspended in half of the starting plasma volume. The procedure was repeated twice in order to produce a platelet concentration four times higher than that of P-PRP.

Platelet count was performed in the PPP, in the pure platelet solution and in the concentrated P-PRP as a quality control.

### Activation of platelet concentrates

P-PRP, PPP, the pure platelet solution and the concentrated P-PRP were activated shortly before use. In order to initiate clotting and trigger the release of platelet content, CaCl_2_ was added at a final concentration of 4.5 mM.

### Bacterial strains

Ten different strains of *E. faecalis*, *S. agalactiae*, *S. oralis* and *S. aureus* respectively, isolated from oral clinical samples, were used. Microorganisms were kept stored at −80°C until use. Bacterial strains were previously identified by biochemical methods (Vitek 2 Compact, bioMériéux, Marcy l'Etoile, France) and confirmed by DNA sequencing of 80 bp of the variable regions V1 and V3 of the 16S rRNA gene by Pyrosequencing (PSQ96RA, Diatech, Jesi, Italy). In addition, ATCC strains were used as controls. Before use, strains were thawed and reconstituted in Brain Heart Infusion broth (BHI; bioMériéux, Marcy l'Etoile, France) for *E. faecalis* and *S. aureus* or BHI additioned with 5% defibrinated blood for *S. agalactiae* and *S. oralis* for 24 hours at 37°C in proper conditions.

### Determination of antibacterial activity

The minimum inhibitory concentration (MIC), defined as the lowest concentration of an antimicrobial substance that inhibits the visible growth of a microorganism, was determined by broth microdilution 2-fold serial method. After seeding on appropriate medium (Trypticase Soy Agar or Columbia Blood Agar; bioMériéux, Marcy l'Etoile, France), a suspension in growth medium (BHI or BHI+5% defibrinated blood) was prepared for each strain, with an optical density equal to 0.5 McFarland (1×10^8^ CFU/ml). After obtaining a concentration of 1×10^4^ CFU/ml using appropriate dilutions, 20 µl of each suspension were inoculated in a 96-wells microplate containing 180 µl of a serial 2-fold dilution (ranging from 1∶2 to 1∶2048) of the material under evaluation. Both CaCl_2_ activated and non activated samples were tested. Positive controls were performed inoculating the microbial suspension in growth medium alone. In order to assess that CaCl_2_ did not possess antimicrobial activity *per se*, controls were performed inoculating microbial suspension in growth medium additioned with CaCl_2_ at a final concentration of 4.5 mM. MIC values, corresponding to the lowest dilution exhibiting no visible bacterial growth, were read visually after 24 hours of incubation at 37°C in proper conditions. The assay was conducted in duplicate for each strain and, if the two MIC values diverged by more than two dilutions, the assay was repeated. MIC values were expressed as dilution from the initial concentration. For each bacterial species and antimicrobial combination, the MIC dilution mode and range were determined.

## Results

### Platelet and leukocyte counts

Platelet concentration in P-PRP pooled blood samples was 2.2 times higher than in whole blood (565×10^3^ platelets/µl and 252×10^3^ platelets/µl, respectively). Platelets in 2-fold and 4-fold concentrated P-PRP were 1.8 and 3.6 times higher than in P-PRP (989×10^3^ platelets/µl and 2034×10^3^ platelets/µl, respectively). Platelets were almost absent in PPP (<10^2^ platelets/µl), while the concentration of the pure platelet solution was almost equal to that of P-PRP (500×10^3^ platelets/µl). Pooled P-PRP was efficiently leukocyte-depleted (<10^3^ white blood cells/µl).

### Determination of antibacterial activity

The MIC values observed for all strains tested ranged between 1∶4 and 1∶16. The MIC modal values for each bacterial species are summarized in Table 1. Both P-PRP and PPP were able to inhibit the growth of *E. faecalis* and *S. aureus* strains at a dilution of 1∶8. *S. agalactiae* and *S. oralis* strains seemed to be slightly more susceptible to P-PRP than PPP, being inhibited by a dilution of 1∶16 for P-PRP and by a dilution of 1∶8 for P-PRP. However, no major differences (>two dilutions) were found between their MIC values. Both 2-fold and 4-fold concentrated P-PRP were able to inhibit the growth of all strains tested at a dilution of 1∶16. In contrast, platelets alone did not show any inhibitory effect at the concentrations tested. Only activated samples displayed an antibacterial activity, whereas non activated samples did not exhibit any effect. CaCl_2_ *per se* did not show any antibacterial activity. MIC values observed for ATCC bacterial strains fell into the same dilution ranges as those of the corresponding clinical isolates.

**Table 1:** Antibacterial activity of platelet concentrates against bacteria isolated from oral cavity.

		MIC (mode)	
		Activated	Non activated
*E. faecalis*	P-PRP	1∶8	no effect
	PPP	1∶8	no effect
	Platelets alone	no effect	no effect
	2-fold concentrated P-PRP	1∶16	no effect
	4-fold concentrated P-PRP	1∶16	no effect
*S. agalactiae*	P-PRP	1∶16	no effect
	PPP	1∶4	no effect
	Platelets alone	no effect	no effect
	2-fold concentrated P-PRP	1∶16	no effect
	4-fold concentrated P-PRP	1∶16	no effect
*S. oralis*	P-PRP	1∶16	no effect
	PPP	1∶4	no effect
	Platelets alone	no effect	no effect
	2-fold concentrated P-PRP	1∶16	no effect
	4-fold concentrated P-PRP	1∶16	no effect
*S. aureus*	P-PRP	1∶8	no effect
	PPP	1∶8	no effect
	Platelets alone	no effect	no effect
	2-fold concentrated P-PRP	1∶16	no effect
	4-fold concentrated P-PRP	1∶16	no effect

MIC values are expressed as dilutions from the initial concentration; in activated samples, CaCl_2_ was added at a final concentration of 4.5 mM. Data are represented as mode obtained from 10 strains of each bacteria.

## Discussion

The regenerative potential of platelet concentrates has been explored considerably during the last two decades. On the contrary, only few reports can be found about their antimicrobial effects in the available literature. To date, the components responsible for the antimicrobial activity of platelet concentrates remain poorly understood. Several antimicrobial factors have been proposed, including platelet antimicrobial proteins and peptides of the innate immune defense, or platelet α-granules components, such as complement and complement-binding proteins [17], [18], [27]–[31]. Direct interaction of platelets with microorganisms, participation in antibody-dependent cell cytotoxicity, release of myeloperoxidase, activation of the antioxidant responsive element and antigen-specific immune response have also been suggested [15], [32]–[33].

The role of leukocytes within platelet concentrates is a matter of intense debate. Some authors have suggested that inclusion of white blood cells in platelet concentrates may help to improve the stability of the scaffold, increase the antimicrobial potential, and help to regulate the inflammatory response [34]. On the contrary, other authors suggested that an additional leukocyte content might increase the inflammatory response at the site because of the metalloproteases, pro-inflammatory proteases and acid hydrolases secreted by white blood cells [35].

Our study was designed to elucidate which components in leukocyte-poor platelet-rich plasma might inhibit bacteria. We tested: 1) P-PRP, that is the formulation commonly used in our clinics, 2) platelets resuspended in PBS, in order to determine the role of platelets isolated from the plasmatic components, and 3) PPP in order to define the antimicrobial role of plasma innate or humoral immune response [36]–[37]. Moreover, we tested the above-mentioned products before and after activation with CaCl_2_, in order to evaluate their antimicrobial activity in the absence of activation of coagulation and release of platelet granules content.

All materials tested were obtained from the same pool of donors in order to limit the impact of individual donor variations.

Products were tested against four different species relevant to be responsible of oral infections which have been previously shown to be susceptible to platelet concentrates activity, such as *E. faecalis*, *S. agalactiae*, *S. oralis* and *S. aureus* [7], [8], [13], [15].

Activated P-PRP inhibited all strains, confirming the observations made in previous research works [7], [8], [13], [15]. Activated PPP showed the same activity of activated P-PRP. However, in the case of *S. oralis* and *S. agalactiae*, a tendency to a greater susceptibility for activated P-PRP was observed, with differences in MIC values equal to 2 dilutions. This trend may be due to the release of platelet-derived antimicrobial proteins, as suggested in a previous work [38]. Finally, platelets alone did not show any antibacterial activity. Taken together, these data seems to suggest that the antimicrobial activity of platelet concentrates against *E. faecalis*, *S. agalactiae*, *S. oralis* and *S. aureus* is sustained by a co-operation of plasma components and platelet-derived factors.

A recent study by Burnouf *et al.* tried to further understand which components in platelet concentrates might bear the antimicrobial activity, testing samples before and after heat complement inactivation [10]. The absence of any bacterial inhibition by any of the heat-treated plasma and platelet materials supported the idea that the plasma complement (and/or other heat-sensitive compounds) played the major role in the bacteriostatic properties of platelet concentrates. We tested heat-inactivated samples too (data not shown), confirming the results of Burnouf *et al.* despite the differences in the experimental design of our study (e.g. different protocol adopted for platelet concentrate production, different method used to evaluate microbial susceptibility, different bacterial strains tested, etc.). These results strengthen the hypothesis of an implication of plasma components in the antimicrobial activity of P-PRP.

We also evaluated the effect of platelet concentration on the antimicrobial activity of platelet concentrate, comparing the activity of 2-fold and 4-fold concentrated P-PRP. We observed that all of them had a similar antibacterial activity against the strains tested, independent of their platelet concentration. These results are consistent with the findings of previous studies showing no correlation between antimicrobial activity and the concentration of platelets in the blood and P-PRP [8], [9].

In addition, we observed that only activated materials were able to inhibit bacterial growth, suggesting that the activation of coagulation is a fundamental step.

Despite the clinical relevance of these results in terms of prevention of infections in surgery settings, future researches should better investigate the potential practical implications of the findings of this study as well as the modality of clinical application of platelet concentrates.
